# The nutritional status of community-dwelling elderly in Lesotho and factors associated with malnutrition

**DOI:** 10.1177/02601060221082368

**Published:** 2022-02-23

**Authors:** Rose Kokui Dufe Turkson, Jennifer Ngounda, Riette Nel, Corinna May Walsh

**Affiliations:** 1 Department of Nutrition and Dietetics, 37702 University of the Free State, Bloemfontein, South Africa; 2 Department of Biostatistics, 37702 University of the Free State, Bloemfontein, South Africa

## Abstract

**Objective:** The elderly living in Africa are prone to malnutrition which is complicated by the high prevalence of poverty. This study assessed the nutritional status of the elderly and factors associated with malnutrition. **Method:** In a cross-sectional survey, the nutritional status of 300 participants aged 65 years and older was determined using the Mini Nutritional Assessment (MNA) questionnaire consisting of 18 questions. Socio-demographic data was obtained using a questionnaire. **Results:** Sixty-six percent were at risk of malnutrition, while 14.6% (n = 44) were malnourished. Participants that did not use electricity as a fuel for cooking versus those that did, had higher odds of being malnourished/ at risk of malnutrition (OR = 1.85 [1.04; 3.31]). Those that did not experience psychological stress or acute disease versus those that did, had lower odds of being malnourished/at risk of malnutrition (OR = 0.33 [0.12; 0.90]). Participants that did not perceive nutritional problems versus those that did, had lower odds of being malnourished/at risk of malnutrition (OR = 0.18 [0.09; 0.34]). Similarly, those that did not perceive their health status as poor versus those that did, had lower odds of being malnourished/at risk of malnutrition (OR = 0.17 [0.08; 0.34]). **Discussion:** The findings indicate that the elderly with more resources, less stress, and better actual and perceived health were less likely to be malnourished. In such communities, routine screening in the elderly is required to identify those with compromised health and nutritional status.

## Introduction

In 2015 about 46 million people aged 60 years and older resided in Sub-Saharan Africa, and it is estimated that by the year 2050 this number will increase to 161 million (United Nations Population Division, 2016). The term “elderly” is often used to describe persons aged 60 years and older (Amarya et al., 2018). The risk of developing nutrition-related disorders is much higher in the elderly population due to their vulnerability, and also the decrease in daily consumption of food with old age (Saghafi-Asl et al., 2018). Malnutrition in the elderly increases the risk of impaired health, quality of life and also leads to increased mortality (Abolghasem Gorji et al., 2017; Ahmed and Haboubi, 2010). As a result of the aging process, the elderly often experience physiological changes such as changes in the nervous system, cognition, memory, and special senses such as taste (Amarya et al., 2018), which may influence food intake. Factors that may contribute to poor nutritional status in elderly persons include; reduced appetite, poor dentition (Chapman, 2006; Lee and Frongillo, 2001), the decline of the gastrointestinal system, chronic diseases, polypharmacy, loneliness, and depression (Morley, 2012; Oliveira et al., 2009). These factors often affect eating patterns and food choices (Broeska et al., 2013).

The elderly aged 65 years and older form 8% of the entire population of Lesotho (International Food Policy Research Institute (IFPRI), 2015; World Food Programme (WFP), 2018). The country faces challenges such as the HIV/AIDS pandemic (25%), widespread poverty (57.1%), and food insecurity (24%), which have a considerable impact on the economy of the country and the health and nutritional status of the inhabitants (United Nations Development Programme (UNDP) and Lesotho Government, 2015; WFP, 2018; IPC, 2019). High levels of malnutrition have been documented throughout the country, especially in children aged under five years, women of childbearing age, and adults (Rothman et al., 2018; The World Bank, 2015). The government of Lesotho provides a non-contributory monthly pension allowance for the elderly aged 70 years and older (International Labour Organisation, 2016). However, studies have shown that, although this pension has had a positive impact on the physical and social needs of the elderly, it is still insufficient, as the funds are often used to support family members, such as grandchildren that are orphaned due to HIV (Croome and Mapetla, 2007; Mugomeri et al., 2017). Consequently, many of the elderly tend to engage in economic activities to improve their livelihoods, which may influence their health and wellbeing. In 2011, 78.9% of the elderly aged between 60 and 80 years were reported to be involved in active economic activities (Ministry of Health (MOH) [Lesotho] and ICF International, 2015).

The Mini Nutritional Assessment (MNA) is a validated nutrition screening tool developed for routine screening of the elderly, to identify and treat nutrition-related problems in a timely manner (Guigoz and Vellas, 1999). It consists of 18 questions that combine to rate anthropometric, dietary, global, and self-view aspects of nutrition. The MNA has proven to be a useful diagnostic tool in the identification of malnutrition in geriatric patients aged 65 years and older, both freely living in the community or institutionalized (Charlton et al., 2007; Inoue and Kato, 2007; Kaiser et al., 2009). In South Africa (a neighbouring country of Lesotho), a malnutrition prevalence of 14.2% was reported in institutionalized elderly patients using the MNA tool, with the prevalence being higher in those residing in a long-term care facility situated in a resource-limited area (Robb et al., 2017).

This research aimed to establish the nutritional status of community-dwelling elderly in urban areas of Maseru Lesotho and to determine the factors associated with malnutrition.

## Methods

### Participants, study site, and sampling

The research had a cross-sectional design and was conducted among the elderly residing in the Maseru district, Lesotho. Maseru district is the national capital of Lesotho and the largest urban area (Bureau of Statistics (BoS), 2007; United Nations Development Programme (UNDP), 2016) situated in the northwest of the country bordering the Free State Province of South Africa. The total population of Maseru is 267 000 (2014) (Central Intelligence Agency (CIA), 2018). To be included in the study, participants had to: 1) be 65 years and older, 2) not intend to relocate during the study period and 3) voluntarily sign informed consent. Bedridden elderly persons and those who were cognitively impaired to such an extent that they could not complete the interview were excluded.

#### Sampling

Maseru is sub-divided into 18 constituencies. For this study, four urban constituencies were randomly selected. From the four selected constituencies, 16 communities were further randomly selected within each constituency. Using a list of all the eligible elderly participants, provided by the prospective community authorities (chiefs), 300 participants were randomly selected using Epi info version 6 from the Center for Disease Control.

### Data collection

Trained fieldworkers (with degrees in nutrition) collected the data. All study participants were interviewed individually on a face-to-face basis, in their home language (English or SeSotho) at their places of residence, after which anthropometric measurements were taken.

### Data collection tools and procedures

Information related to socio-demographic status such as age, gender, educational level, employment status, and living conditions (access to drinking water and electricity) were collected using a questionnaire adapted from the “Assuring Health in the Free State” (AHA-FS) study, (Walsh and Van Rooyen, 2015). The MNA tool was used to obtain data on the nutritional status of the elderly. The tool consisted of 18 questions grouped into four sections namely: anthropometric measurements, dietary assessment, a global evaluation, and a subjective evaluation (Guigoz, 2006).

### Anthropometric measurements

For anthropometric assessment, weight, height, mid-arm circumference (MAC), and calf circumference (CC) relevant to the elderly population were all measured. All anthropometric measurements were taken in duplicate using standardized techniques (WHO, 1995), and an average of the two measurements was calculated. Weight and height were measured to determine body mass index (BMI). The elderly were measured wearing minimal clothing, and barefoot. Weight was measured using a high-capacity electronic flat scale (Seca 813, Germany) and recorded to the nearest 0.1 kg. When necessary, support was provided for the participants to mount the scale with ease. Height was measured using a rigid stadiometer and recorded to the nearest 0.1 cm. For participants who were too weak to stand, calculated weight and height using knee height and mid-upper arm circumference were used. To measure knee height, the participant had to bend the knee and ankle of one leg at a 90-degree angle while sitting on a chair with legs on the floor. A non-stretchable tape measure was placed under the heel of the foot in line with the ankle bone. Measurements were recorded to the nearest 0.1 cm (Nestle Nutrition Institute (NNI), 2011). BMI values were grouped into four categories on the MNA questionnaire BMI < 19 kg/m^2^; BMI 19 kg/m^2^ to < 21 kg/m^2^; BMI 21 kg/m^2^ to < 23 kg/m^2^; and BMI > 23 kg/m^2^. Mid arm circumference (MAC) was measured to the nearest 0.1 cm on the non-dominant arm, at the midpoint between the acromion and the olecranon. MAC was categorized as MAC<21cm; MAC 21 to 22cm and MAC>22cm. Calf circumference (CC) was measured to the nearest 0.5 cm, at the largest circumference of the calf, with the knee and ankle bent to a 90-degree angle, using a non-stretchable tape measure (Charlton et al., 2007; NNI, 2011). CC was categorized as CC<31cm and CC>31cm. To obtain an indication of perceived weight loss in the past 3 months, participants were asked if their clothing or belt size had changed.

#### Dietary assessment

The dietary assessment included eight questions related to the number of full meals consumed daily (defined as a meal that includes two or more different items); daily servings of dairy; weekly servings of beans or eggs; daily consumption of meat, fish, or poultry; daily servings of fruits or vegetables; food intake declined over the past three months due to loss of appetite, digestive problems, chewing or swallowing difficulties; the daily fluid intake; and the need for feeding assistance.

#### Global evaluation

The global evaluation consisted of six questions related to participants living conditions (living independently); prescription drug use; the presence of psychological stress or acute disease; mobility; neuropsychological problems and the presence of pressure sores or ulcers.

#### Subjective assessment

The subjective assessment included questions that enabled participants to express their self-view of nutritional status and how participants perceived their health status in comparison to their peers.

#### Total MNA^®^ score

Scores obtained in each of the above assessments were summed to achieve a total MNA score. Anthropometric assessments contributed 8 points to the total MNA scores, whereas dietary, global, and subjective assessments contributed 9 points, 9 points, and 4 points respectively. The maximum achievable total MNA score was 30 points (ranging from 0 to 30). The total MNA score was interpreted as follows to indicate malnutrition: MNA score <17 points = malnutrition; MNA score 17 to 23.5 points = at risk of malnutrition; and 24 to 30 points = normal nutritional status (well-nourished) (Guigoz and Vellas, 1999).

### Pilot study

A pilot study was conducted on 10 elderly participants from the selected communities to determine whether the questions in the questionnaire were well understood by participants and to establish the average time needed to complete the questionnaire and anthropometric measurements. No changes were made to the questionnaire after the pilot; therefore, the data was included in the main research.

### Statistical analysis

Data were analyzed using the SAS software, Version 9.4 of the SAS System for PC. Copyright © 2018 SAS Institute Inc.. SAS and all other SAS Institute Inc. product or service names are registered trademarks or trademarks of SAS Institute Inc., Cary, NC, USA. Descriptive statistics, namely frequencies and percentages were calculated for categorical data. The distribution of numerical data was checked therefore the summary statistics medians and percentiles were used. The MNA classifications of malnutrition and at risk of malnutrition were grouped together and compared to the well-nourished group. The two groups were assessed to establish their relationship with socio-demographic and nutritional variables using Chi-square or Fisher's exact test for categorical data. A backward stepwise logistic regression to model malnutrition/at risk of malnutrition controlling for age and gender regarding socio-demographic factors was done. The odds ratios, 95%CI, and *p*-values were calculated for the risk factors in the final model. The following variables were included in the model (based on the fact that there were significant associations between these variables and malnutrition/at risk of malnutrition): gender, type of fuel used for cooking, perceived weight loss >3kg, psychological stress/acute disease, perceived nutritional problems, and perceived poor health status. A *p*-value of <0.05 was considered statistically significant and these variables were then included in the model.

### Ethical considerations

The research was approved by the Health Sciences Research Ethics Committee of the University of the Free State, South Africa (ECUFS NR 217/2014). Approval was also granted by the Ethics Committee of the National Institution Review Board of the Ministry of Health, Lesotho (ID71-2015). A site map, together with a list of the elderly aged 65 years and older were used to locate and recruit participants. The study was explained by a field worker to all participants in a preferred language (English or Sesotho). In the case of illiterate participants, a thumbprint was used as a signature for consent. Participants were informed that participation in the study was voluntary and that all the information obtained would be kept strictly confidential.

## Results

### Participants’ characteristics and household data

A total of 300 elderly participants were included in the study. Of these, 87 (29.0%) were male and 213 (71.0%) were female. The median age was 74 years (range: 64-95 years). Table 1 depicts data on the socio-demographic and household profile of the study population. Fifty-six percent of the elderly had been residing in the urban area for over 41 years. Those with primary school education were 65.5%, and 6.3% had obtained tertiary education. Those widowed were 61.0%. Unemployment was 43.1% and 46.9% of the participants were pensioners. A monthly household income ranging from 100 to 1000 Lesotho Maloti (approximately US$8.3 to US$83) was reported by 73.5% of elderly participants; and in 96.3% of the households, an average of three people contributed to this income. Furthermore, 70% of the participants reported the income to be the same in the 6 months prior to the research being conducted. Results on basic household amenities indicated that 69.2% had electricity, and 77.3% had water from their own tap. The pit latrine (61.7%) was the most commonly used toilet facility. Gas (44.1%) was the main source of fuel for cooking in most households. Table 2 shows that there were significant differences between the well-nourished and the malnourished groups with regards to the type of toilet (p < 0.01) the type of fuel used for cooking (p < 0.01) and the source of drinking water (p = 0.04).

**Table 1.** Socio-demographic and household profile of elderly participants (*n* = 300).

	*n*	%
**Age category (years)**		
65-69	84	28.8
70–74	81	27.0
75–79	66	22.0
80–84	37	12.3
85 +	32	10.7
**Gender**		
Males	87	29
Females	213	71
**Number of years residing in urban area**		
>10 years	32	10.7
10–20	29	9.7
21–30	23	7.6
31–40	47	15.7
41 +	169	56.3
**Marital status**		
Never married	17	5.6
Currently married	48	16.0
Living with partner	46	15.3
Widowed	183	61.0
Separated	3	6.3
Divorced	3	0.3
**Level of education**		
None	35	11.7
Primary school	197	65.8
Secondary school	47	15.7
Tertiary	19	6.3
Don’t know	1	0.3
**Occupation (n = 299)**		
Unemployed	129	43.1
Self-employed	21	7.0
Full-time wage earner	9	3.0
Pension^a^	140	46.9
**Household income** ^b^ **(n = 298)**		
None	13	4.4
100–500	138	46.3
501–1000	81	27.2
1001–3000	43	14.4
3001–5000	3	1.0
Over 5000	7	2.3
Don’t know	13	4.4
**Number of people contributing to household income**		
0–3	289	96.3
4–8	11	3.7

^a^ Monthly household income in Lesotho Maloti (M); M1 is approx. $0.08.

^b^ Includes non-contributory old age pensions & normal pensions.

**Table 2.** Associations of basic household amenities with nutritional status in the elderly.

	Well-nourished *n* (%)	Malnourished	*P*-value^*^
**Type of dwelling (n = 299)**			
Brick, concrete	52 (91.2)	191 (78.9)	0.35
Traditional mud	1 (1.8)	10 (4.1)	
Tin	0	8 (3.3)	
Plank, wood	0	1 (0.4)	
Other	4 (7.0)	32 (13.2)	
**Electricity (n = 299)**			
Yes	49 (84.5)	158 (65.6)	**<0.01**
No	9 (15.5)	83 (34.4)	
**Type of toilet (n = 300)**			
Flush	15 (25.9)	19 (7.8)	**<0.01**
Pit	25 (43.1)	160 (66.1)	
Bucket ,pot	16 (27.6)	50 (20.7)	
VIP	2 (3.4)	13 (5.4)	
**Fuel for cooking (n = 299)**			
Electric	23 (39.7)	56 (23.2)	**<0.01**
Gas	31 (53.5)	101 (41.9)	
Paraffin	3 (5.2)	64 (26.6)	
Wood, coal	1 (1.7)	10 (4.1)	
Sun	0	5 (2.1)	
Open fire	0	5 (2.1)	
**Source of drinking water (n = 300)**			
Own tap	53 (91.3)	179 (73.9)	**0.04**
Communal tap	2 (3.5)	38 (15.7)	
River, dam	0	4 (1.7)	
Borehole, well	1 (1.7)	12 (5.0)	
Other sources	2 (3.5)	9 (3.7)	
**Household appliances**			
Working Refrigerator (n = 298)			
**Yes**	40 (69.0)	105 (43.8)	**<0.01**
**No**	18 (31.0)	135 (56.2)	
Working Stove (gas/ electricity) (n = 298)			
**Yes**	55 (94.8)	165 (68.7)	**<0.01**
**No**	3 (5.2)	75 (31.3)	
Working Primus/paraffin stove (n = 298)			
**Yes**	27 (46.5)	163 (67.9)	**<0.01**
**No**	31 (53.5)	77 (32.1)	
Working microwave (n = 298)			
**Yes**	28 (48.3)	59 (24.6)	**<0.01**
**No**	30 (51.7)	181 (75.4)	

*P-value for Chi-square or Fisher's exact test for categorical data. Significance set at *p* < 0.05.

### Mini nutritional status assessment -MNA^®^

#### Anthropometric assessment

The median BMI of participants was 25.2 (14.5–46.1) kg/m^2^. More than half (63.6%) had a BMI greater than 23 kg/m^2^, and 14% had BMI less than 19 kg/m^2^. Calf circumference measurements showed that 88.7% had CC ≥33 cm, and results for MAC revealed that almost all the participants (96.7%) had MAC greater than 22 cm. Finally, 40.3% perceived no weight loss during the past three months. In Table 3, it is evident that there were significant differences between the well-nourished and the malnourished groups in terms of BMI (p < 0.01), calf circumference (p < 0.01) and perceived weight loss (p < 0.01).

**Table 3.** Anthropometric assessments and associations with nutritional status in the elderly (n = 300).

	Well-nourished *n* (%)	Malnourished	*P*-value^‡^
***BMI (kg/m^2^)***	28.6 (26.1–32.3)*	23.8 (20.6–28.8)*	**<0.01**
< 19	2 (3.5)	40 (16.5)	**<0.01**
19 to < 21	0	29 (11.9)	
21 to <23	1 (1.7)	37 (15.3)	
≥ 23	55 (94.8)	136 (56.2)	
***Mid Arm circumference (MAC) (cm)***			
< 21	0	6 (2.5)	0.66
21 to 22	0	4 (1.6)	
>22	58 (100)	232 (95.9)	
***Calf circumference (CC)(cm)***			
< 31	0	34 (14.0)	**<0.01**
≥ 31	58 (100)	208 (86.0)	
***Perceived weight loss in last 3 months^a^***			
Weight loss during last 3 months	2 (3.4)	50 (20.7)	**<0.01**
Does not know	2 (3.4)	61 (25.2)	
Weight loss between 1 and 3 kg	7 (12.1)	57 (23.5)	
No weight loss	47 (81.0)	74 (30.6)	

^a^ Participants were asked if their clothing size/belt size had changed.

^‡^ *P-*value for Kruskal-Wallis test for numerical data and Chi-square or Fisher's exact test for categorical data. Significance set at p < 0.05.

*All such values are median (interquartile range).

#### Dietary assessment

Participants that consumed three full meals daily were 62.3%. Fifty-three percent reported to consume none or just one type of protein-rich food daily, while 69.3% reported daily consumption of two or more servings of fruit and vegetables. Almost all the elderly participants self-fed without any problem (99.0%); however, 50.1% reported a loss of appetite in the past three months, with 11.3% having severe appetite loss, whilst 38.8% had moderate appetite loss. A significantly higher percentage of elderly in the well-nourished group consumed three full meals daily, consumed more protein-rich foods, and more fruits or vegetables compared to the malnourished group; (p < 0.01) (Table 4). Furthermore, a significantly higher percentage of elderly participants in the well-nourished group did not suffer appetite loss compared to the malnourished group (p < 0.01) (Table 4).

**Table 4.** Dietary assessment factors and associations with nutritional status in the elderly (*n* = 300).

	Well-nourished *n* (%)	Malnourished	*P*-value*
**Full meals eaten per day (n = 299)^a^**	** **	** **	** **
1 meal	0	6 (2.5)	**<0.01**
2 meals	8 (13.8)	99 (41.1)	
3 meals	50 (86.2)	136 (56.4)	
***Consumption of dairy, beans/egg, meat etc.***			
0 or yes to 1	15 (25.9)	145 (59.9)	**<0.01**
Yes to 2	23 (39.7)	69 (28.5)	
Yes to 3	20 (34.5)	28 (11.6)	
***Consume 2 or more serving of fruits or vegetables daily***			
No	7 (12.1)	85 (35.1)	**<0.01**
Yes	51 (87.9)	157 (64.9)	
***Daily fluid consumption (water, juice, coffee, tea, milk, wine) (n* *=* *299)***			
Less than 3 glasses	5 (8.6)	30 (12.5)	**<0.01**
3 to 5 glasses	11 (19.0)	69 (28.6)	
More than 5 glasses	42 (72.4)	142 (58.9)	
***Need for feeding assistance (n* *=* *299)***			
Unable to eat without assistance	0	0	0.52
Self-fed with some difficulties	0	3 (1.2)	
Self-fed without any problem	58 (100)	238 (98.8)	
***Declining food intake over the past three months (n* *=* *299)***			
Severe loss of appetite	0	34 (14.1)	**<0.01**
Moderate loss appetite	8 (13.8)	108 (44.8)	
No loss of appetite	50 (86.2)	99 (41.1)	

^a^ A meal that includes two or more different items.

*P-value for Chi-square or Fisher's exact test for categorical data. Significance set at *p* < 0.05.

#### Global evaluation

Elderly participants residing independently were 80.7%. Thirty percent reported using more than three medications daily, and only 4.4% were physically immobile. Only 7.3% of participants reported pressure sores. Psychological stress was reported in 25.0% and 46.3% reported experiencing some form of cognitive impairment. A significantly higher percentage of the elderly in the well-nourished group lived independently compared to the malnourished group (p = 0.03) (Table 5). Moreover, significantly more elderly participants in the malnourished group experienced neuropsychological problems compared to those in the well-nourished group (p < 0.01) (Table 5).

**Table 5.** Global and subjective assessments and associations with nutritional status in the elderly (n = 300).

	Well-nourished *n* (%)	Malnourished	*P*-value*
***Global assessment***			
**Live independently**			
No	5 (8.6)	53 (21.9)	**0.03**
Yes	53 (91.4)	189 (78.1)	
**Take more than 3 prescription drugs daily**			
No	34 (58.6)	174 (71.9)	0.06
Yes	24 (41.4)	68 (28.1)	
**Suffered any psychological stress/acute disease**			
No	41 (70.7)	184 (76.0)	0.40
Yes	17 (29.3)	58 (24.0)	
**Mobility**			
Bed bound	0	4 (1.7)	0.13
Able to get out of bed/chair but does not go out	1 (1.7)	8 (3.3)	
Goes out	57 (98.3)	230 (95.0)	
**Neuropsychological Problem**			
Severe dementia	0	23 (9.5)	**<0.01**
Mild dementia	15 (25.9)	101 (41.7)	
No psychological problems	43 (74.1)	118 (48.8)	
**Pressure sores/skin ulcers**			
Yes	3 (5.2)	19 (7.8)	0.59
No	55 (94.8)	223 (92.2)	
***Subjective assessment***			
**Perceived Health status compared to others (n = 299)**			
Not as good	2 (3.4)	79 (32.8)	**<0.01**
Does not know	2 (3.4)	34 (14.1)	
As good	24 (41.4)	91 (37.8)	
Better than others	30 (51.7)	37 (15.3)	
**Perceived Nutritional problem**			
Major malnutrition	1 (1.7)	80 (33.2)	**<0.01**
Does not know or moderate malnutrition	11 (19.0)	90 (37.3)	
No nutritional problem	46 (79.3)	71 (29.5)	

*P-value for Chi-square or Fisher's exact test for categorical data. Significance set at *p* < 0.05.

#### Subjective assessment

Results relating to “self-view” on health and nutritional status revealed that 60.8% of the elderly participants perceived their health status to be either “as good” or “better” as compared to others, 27.0% perceived themselves to have a major nutritional problem. Lastly, 39.3% expressed that they did not have any nutritional problems. Significantly more elderly participants in the malnourished group perceived poor health and nutritional status compared to the well-nourished group (p < 0.01) (Table 5).

#### Total MNA^®^ score

The median MNA score was 20.5 (range 9.0–28.0). The final scores obtained for each of the four sections included in the MNA revealed that 66.0% (n = 198) of the elderly participants were at risk of malnutrition, 14.6% (n = 44) were malnourished and 19.4% (n = 58) were well-nourished.

Logistic regression analysis (Table 6) showed that being male increased the odds of being malnourished/at risk of malnutrition (OR = 2.55 [1.01; 6.43]). Participants that did not use electricity as a fuel for cooking versus those that did, had higher odds of being malnourished/ at risk of malnutrition (OR = 1.85 [1.04; 3.31]). Those that did not perceive weight loss >3kg versus those that perceived weight loss >3kg had lower odds of being malnourished/at risk of malnutrition (OR = 0.27 [0.16; 0.46]). Those that did not experience psychological stress or acute disease versus those that did, had lower odds of being malnourished/at risk of malnutrition (OR = 0.33 [0.12; 0.90]). Participants that did not perceive nutritional problems versus those that did, had lower odds of being malnourished/at risk of malnutrition (OR = 0.18 [0.09; 0.34]). Similarly, those that did not perceive their health status as poor versus those that did, had lower odds of being malnourished/at risk of malnutrition (OR = 0.17 [0.08; 0.34]).

**Table 6.** Factors associated with malnutrition or at risk of malnutrition: logistic regression.

Variable	Description	Odds ratio (95% CI)	*P*-value
Gender	male vs. female	2.55 (1.01; 6.43)	0.05
Type of fuel used for cooking	did not use electricity vs. used electricity	1.85 (1.04; 3.31)	0.04
Perceived weight loss >3kg	No vs. Yes	0.27 (0.16; 0.46)	<0.01
Psychological stress/acute disease	No vs. Yes	0.33 (0.12; 0.90)	0.03
Perceived nutritional problems	No vs. Yes	0.18 (0.09; 0.34)	<0.01
Perceived poor health status	No vs. Yes	0.17 (0.08; 0.34)	<0.01

## Discussion

This study population was characterized by suboptimal living conditions, and poor nutritional status, as determined by the overall MNA score. The findings revealed that a large percentage of the elderly residing in urban Maseru were generally at risk of malnutrition. Previous research in Lesotho has reported malnutrition to be a common problem affecting the elderly, both institutionalized and community-dwelling (Ministry of Social Development (MSD), 2014; Sello et al., 2018). In Lesotho, the causes of malnutrition in the elderly have been attributed to both economical and physiological circumstances. Elderly persons tend to be food insecure due to a lack of money to purchase food (MSD, 2014). Moreover, those involved in subsistence farming face challenges of poor food production due to adverse weather conditions, pests, lack of fertilizers, and lack of high-yield seeds (WFP, 2018). Previous outcomes from focus group discussions amongst the elderly in Lesotho showed that this group suffered from physiological problems such as dental problems, sensory losses, and loss of appetite due to chronic medication intake, which hindered food intake. Furthermore, at times, the incapability to cook also contributed to the elderly skipping meals, therefore posing risk for malnutrition (MSD, 2014). Most of the participants in this study resided independently and often had to prepare their food. Moreover, we observed that a larger percentage of the well-nourished elderly resided independently compared to the group of malnourished participants. Previous studies in developing countries have reported factors such as old age, being widowed, illiteracy, low income, duration of stay in the nursing home, number of diseases, number of medications taken, anthropometric data, and dentition and vision problems to significantly affect the nutritional status of the elderly, both institutionalized and community-dwelling (Hallaj, 2015; Boulos et al., 2014).

In this study, males were more likely to be malnourished than females. Having basic household amenities such as electricity, own tap for water, and appliances that are essential for food processing and storage were associated with better nutritional status. Moreover, participants that reported using an electric stove for cooking had lower odds of being malnourished or at risk of malnutrition. Although the results from the dietary assessment show that most participants consumed three full meals daily, a limitation of this study was that the quantity and quality of these meals were not assessed, and may have been largely inadequate, resulting in the high percentage of elderly participants that were malnourished/at risk for malnutrition.

Despite the high levels of malnutrition and risk of malnutrition in this study population, 63.6% of elderly participants had BMI greater than 23 kg/m^2^. BMI is known to normally increase with advancing age (Babiarczyk and Turbiarz, 2012; Mathus-Vliegen, 2012), and therefore the relatively high percentage of elderly with higher BMI observed in this research is not unexpected. With advancing age, the use of BMI may not be an appropriate tool for identifying poor nutritional status because of the changes in body composition and reduced body height. A BMI between 25.0 kg/m^2^ and 29.9 kg/m^2^ should be considered desirable in the elderly population (Kyle et al., 2002).

Malnutrition and the risk of malnutrition are common in the elderly that are cognitively impaired (Isaia et al., 2011; Roque et al., 2013). The results in this study showed that the percentage of elderly that had some form of dementia was significantly higher in the malnourished group compared to the well-nourished group. Amongst community-dwelling elderly, the risk of malnutrition has been previously associated with depression, poor cognition, and difficulty in meal preparation (Iizaka et al., 2008; Suzana et al., 2013; Naidoo et al., 2015). In the current study perceived “poor” health and nutritional status were higher in participants that were malnourished. These findings are consistent with previous research conducted on the elderly (Chavarro-Carvajal et al., 2015).

## Conclusion

To our knowledge, this is the first study to assess the nutritional status of the elderly in Lesotho using the MNA tool. The results showed that the occurrence of malnutrition and the risk of malnutrition in the elderly was generally high. The findings seem to indicate that the elderly with more resources, less stress, and better actual and perceived health were less likely to be malnourished. This is evidenced by the result that using electricity as a fuel for cooking increased the odds of being well-nourished in elderly participants. Furthermore, participants that did not experience psychological stress or acute disease had lower odds of being malnourished/at risk of malnutrition compared to those that did, while, having a poor self-perception of health and nutritional status increased the odds of malnutrition or being at risk of malnutrition. Further research in similar settings is required to establish the impact of relevant and culturally acceptable interventions that aim to address poverty, psychological stress, poor health, and the nutritional wellbeing of the elderly. The current findings can advise interventions targeted at the elderly, to ensure timely diagnoses and management of malnutrition. In developing countries, where living conditions are often suboptimal, there is a need for routine nutritional screening in the elderly to identify those with compromised health and nutritional status that can benefit from intervention.

## Ethical considerations

The research was approved by the Health Sciences Research Ethics Committee of the University of the Free State, South Africa (ECUFS NR 217/2014). Approval was also granted by the Ethics Committee of the National Institution Review Board of the Ministry of Health, Lesotho (ID71-2015). A site map, together with a list of the elderly aged 65 years and older were used to locate and recruit participants. The study was explained by a field worker to all participants in a preferred language (English or Sesotho). In case of illiterate participants, a thumbprint was used as a signature for consent. Participants were informed that participation in the study was voluntary and that all the information obtained would be kept strictly confidential.
